# Editorial: Intrinsically Disordered Proteins and Regions: The Challenge to the Structure-Function Relationship

**DOI:** 10.3389/fmolb.2022.962643

**Published:** 2022-07-06

**Authors:** Angelo Toto, Pietro Sormanni, Cristina Paissoni, Vladimir N. Uversky

**Affiliations:** ^1^ Istituto Pasteur-Fondazione Cenci Bolognetti, Dipartimento di Scienze Biochimiche “A. Rossi Fanelli” and Istituto di Biologia e Patologia Molecolari del CNR, Sapienza Università di Roma, Rome, Italy; ^2^ Yusuf Hamied Department of Chemistry, University of Cambridge, Cambridge, United Kingdom; ^3^ Dipartimento di Bioscienze, Università degli Studi di Milano, Milano, Italy; ^4^ Department of Molecular Medicine and Byrd Alzheimer’s Research Institute, Morsani College of Medicine, University of South Florida, Tampa, FL, United States

**Keywords:** intrinsically disordered protein, intrinsically disordered region, structure-function continuum, protein-protein interaction, multifunctional protein, protein structure

Proteins are the bricks of life and the major working horses of the cell, as they serve as crucial building blocks forming the basic framework of the cell cytoskeleton and carry out most of the biochemical functions inside and outside cells. For more than one hundred years, proteins were mostly considered through the paradigm of the sequence-structure-function relationship, where the amino acid sequence determines a uniquely folded 3D structure that in turn defines protein function (Petsko and Ringe, 2004). This paradigm grew up from the classical lock-and-key model, where the specific complementarity of the geometric shapes of the enzyme and the substrate provides their exact fit defining the specificity of the enzyme-substrate interaction (Fischer, 1894; Lemieux and Spohr, 1994). Recent years, however, convincingly underscored the crucial importance of protein dynamics and structural flexibility. An extreme case of such flexibility is given by intrinsically disordered proteins (IDPs), which lack defined three-dimensional structures, while retaining functions (Wright and Dyson, 1999; Uversky et al., 2000; Dunker et al., 2001; Tompa, 2002). However, more often, proteins alternate stably folded parts and intrinsically disordered regions (IDRs) (Dunker et al., 2000; Ward et al., 2004; Oldfield et al., 2005; Xue et al., 2012; Peng et al., 2014). Importantly, different parts of a protein molecule can be (dis)ordered to different degree. As a result, the structure of almost any protein is characterized by high spatiotemporal heterogeneity, and instead of being a rigid, crystal-like entity, a protein molecule exists as a highly dynamic conformational ensemble, where different local minima may possess different functional potentials (Uversky, 2015). This indicates that the molecular roots of the protein (multi)functionality—which cannot be easily explained by classic lock-and-key representation—are best described by a more general “protein structure-function continuum” model, where a protein exists as a dynamic conformational ensemble containing multiple different forms characterized by a broad spectrum of structural features and possessing different functional potentials (Uversky, 2015; Uversky, 2016). Furthermore, many IDPs/IDRs can undergo liquid-liquid phase separation (LLPS) leading to the formation of the phase separated liquid droplets and thereby controlling the biogenesis of the membrane-less organelles, which are abundantly present in living cells, have multiple crucial functions, and play important roles in the spatio-temporal organization of the intracellular space (Antifeeva et al., 2022). Finally, given the well-recognized link between protein disorder and biological function and dysfunction, characterizing the dynamics of IDPs and IDRs is of fundamental importance for complete comprehension of the mechanisms by how these proteins exert their functions.

This Research Topic includes seven studies dedicated to the different aspects of the normal and pathological functions of several IDPs and IDRs.

Rodriguez Camargo et al. investigated aggregation mechanisms of the human islet amyloid polypeptide (IAPP). This short intrinsically disordered protein, being associated with diabetes type II, is among the most amyloidogenic peptide hormones known. The use of global chemical kinetic analyses connecting the macroscopic measurements of aggregation to the microscopic mechanisms revealed that the toxic IAPP oligomers are generated *via* the secondary nucleation catalyzed by the fibril surface. Furthermore, in experiments, where cells were treated by samples from different time points of the IAPP aggregation process, the maximal cellular dysfunction was reached when the population of the IAPP oligomeric intermediates was maximal. This suggests that most effective therapeutic candidates for diabetes type II may be found among compounds targeting secondary nucleation of IAPP, to inhibit the formation of oligomers.

Salem et al. discussed the roles of intrinsic disorder in a highly conserved nuclear matrix DNA/RNA-binding protein Matrin3 with multiple important functions, such as management of chromosomal distribution, modulation of alternative splicing, regulation of the mRNA nuclear export and binding and stabilizing mRNA. Mutations in Matrin3 are associated with familial amyotrophic lateral sclerosis (fALS), and its cellular mislocalization is found in sporadic ALS (sALS). This protein can undergo LLPS, the efficiency of which is regulated by various posttranslational modifications, and has an extensive interactome that overlaps with those of TDP-43 and FUS.

Ren et al. dedicated their study to the analysis of the effect of the congenital cataract-associated mutation S93R on the structure and stability of βB1-crystallin, which is the most abundant water-soluble protein in mammalian lens with an essential role for lens transparency. Both the N- and C-terminal arms of this protein (residues 1–60 and 230–252) are disordered, but play crucial roles in homo- and heteromolecular interactions (Dolinska et al., 2009; Srivastava et al., 2009). Using a broad set of experimental and computational tools, the authors showed that the S93R mutation causes lower structural stability and solubility, increases sensitivity to environmental stress, enhances aggregation propensity, and impairs cellular viability.

De Simone et al. analyzed the peculiarities of the membrane binding by α-synuclein (αS), which is a highly studied IDP, whose aggregation is associated with Parkinson’s disease. αS forms a fuzzy complex with membranes, where, in addition to acquiring α-helical structure, noticeable levels of disorder are retained. The authors used enhanced coarse-grained MD simulations to look at the details of the double-anchor membrane binding by the αS central region (residues 65–97), which is believed to promote clustering of synaptic vesicles.

Tashiro et al. conducted detailed structural characterization of a 79-residue-long Int8 domain of the alternatively spliced variant of HER2, herstatin. One peculiar feature of this domain with strong tumor-suppressive activity is that it is encoded by an intron (int-8). The authors showed that that the isolated Int8 domain is mostly disordered but contains some low levels of helical structure.

Micsonai et al. elaborated an automatized binary disorder–order classification method based on the analysis of the far-UV circular dichroism (CD) spectroscopy data. CD is a popular technique for low resolution structural analysis of proteins, with far-UV CD spectra containing information mostly on protein secondary structure. Since IDPs are characterized by specific shapes of their far-UV CD spectra, the authors searched for an optimal method to identify IDPs from the spectral information gathered by CD spectroscopy. The proposed method can be used to detect “global” disorder; i.e., it identifies highly disordered proteins with coil-like and pre-molten globule-like properties.

Finally, Rowling et al. investigated the multivalent interactions between the β-catenin, which is an Armadillo (ARM) repeat-protein from the Wnt signaling pathway, and its intrinsically disordered partner, adenomatous polyposis coli (APC), which is a giant multivalent scaffold. The resulting complex brings together the different components of the “β-catenin destruction complex.” Peculiar feature of APC is the presence of long IDR with multiple short regions responsible for β-catenin bonding. The authors analyzed the multivalent interactions between β-catenin and the highest affinity APC repeat using equilibrium and kinetic approaches. They also dissected the peculiarities of the molecular recognition utilizing a combination of single-site substitutions, deletions, and insertions.
